# Biphasic behavior of T cell subsets reflects failure of early anti-myeloma response and leads to progressive T cell dysfunction

**DOI:** 10.1016/j.neo.2025.101208

**Published:** 2025-07-11

**Authors:** Suchita Suryakant Jadhav, Vipin Sharma, Aharon Lion, Lasser-Katz Efrat, Iftach Shaked, Galia Luboshits, Michael A. Firer

**Affiliations:** a Dept. Chemical Engineering & Biotechnology, Ariel University, Ariel 40700, Israel; b Adelson School of Medicine, Ariel University, Ariel, 40700, Israel

**Keywords:** Multiple myeloma, Immune profile, mouse model, MGUS, SMM, T cell exhaustion, T effector cells

## Abstract

**Introduction:**

Multiple Myeloma (MM) progresses over 2-3 decades through two pre-malignant stages (MGUS and SMM), culminating in clinically active disease. Given the limitations in acquiring sequential bone marrow (BM) samples from patients over this time frame, the mechanisms that compromise immunosurveillance and promote the development of MM remain

**Methods:**

Balb/c mice inoculated with MOPC315.BM myeloma cells were followed over the next 220 days. Blood and bone marrow samples were collected on days 80, 150, and 220 post cell inoculation. Blood samples were used to monitor levels of paraprotein and whole blood cell counts. BM aspirates were used for deep immune profiling by flow cytometry and for T cell function assays.

**Results:**

Blood analyses validated that the model reflects serological features of human MM. Analysis of BM samples revealed a biphasic behavior of T regulatory cells, Th17 cells, CD8+ cytotoxic T cells and NK cells, as well as skewing of CD4+ and CD8+ T memory cell subset distributionss, suggesting failure of an early anti-myeloma response, which is replaced by progressive immunosuppression, and dysfunction and exhaustion of CD8+ T cell tumor cytotoxicity.

**Conclusion:**

Our new model is a flexible tool to investigate the early cellular interactions that initiate immunosuppression and MM disease progression. The model can also be used to test the efficacy of new therapeutic strategies.

## Introduction

Multiple Myeloma (MM) is an incurable malignancy with a median Overall Survival (OS) of about 8 years. MM probably begins in the 3-5 decade of life as an asymptomatic precursor condition known as Monoclonal Gammopathy of Undetermined Significance (MGUS) in which a transformed plasma cell clone establishes a niche in the bone marrow (BM). A small percentage of individuals with MGUS slowly progress to another asymptomatic intermediate stage known as Smoldering Multiple Myeloma (SMM) and only some of these patients eventually develop clinically overt MM [[1], [2], [3]].

The proliferation of the initial myeloma clone largely depends on its interaction with auxiliary cells in the BM and its ability to suppress immune attack [4,5]. A better understanding of these interactions may highlight the mechanisms responsible for tumor escape, breakdown in immunosurveillance, and disease progression and lead to novel immune-based therapeutic interventions [6,7]. However, given the asymptomatic nature of MGUS and SMM, and the extended time frame of 2-3 decades for the development of clinically active MM, tracking early alterations in the immune microenvironment in sequential BM samples from the same patients becomes untenable. This leaves researchers with the sub-optimal alternative of comparing cohorts of different patients at different stages of MM progression. These studies have, nonetheless, led to some insights into the underlying immune suppression that seems to accompany MM disease progression [8,9]. For example, most, but not all [10] studies report that CD4+ T regulatory cell (Treg) frequencies are normal at the early MGUS stage, [11]but appear to be elevated in clinically active MM [12,13] and there is also evidence that effector T-memory cells become exhausted with disease progression [14].

The limitation in acquiring sequential BM samples from patients can be partially addressed by manipulating validated MM mouse models that have already provided useful translational data. One example is the model developed by Bogen and colleagues in which inoculation BM-homing MOPC315.BM cells in immunocompetent Balb/c mice induces osteolytic and immunological features that mirror human MM [15]. We recently recalibrated this model to reflect the early stages of the disease [16]. Here, we expand on those results and report a detailed quantitative analysis of changes in biochemical, hematological and immune profiles and functional parameters for up to 220 days in mice inoculated with only a small number of MOPC315.BM myeloma cells. The data show that the model reflects the hematological and biochemical parameters of human MM and highlights the breakdown in immune surveillance in the early stages of the disease. We also discuss how the model can be used for new basic studies on the early pathogenesis of MM and for pre-clinical studies to test novel treatments to overcome immune exhaustion and inhibit the progression of the disease at its early stages.

## Materials and methods

### Mice and early-to-late multiple myeloma model (ELMM)

All animal experiments were carried out following a protocol approved by the Ariel University Institutional Animal Care and Use Committee (AU IL 22040-116). This permit also required the removal of animals from the experiment if they developed designated signs of morbidity. The PS software package from Vanderbilt University calculated that to test the null hypothesis of no difference between groups under statistical conditions of α = 0.05 and *P* = 0.8, sampling groups need to consist of 5 mice each. Male Balb/c (H-2d) mice of 9-12 weeks of age were purchased from Envigo Laboratories in Jerusalem, Israel. MOPC315.BM cells secreting an IgA paraprotein were kindly provided by Prof. Bjarne Bogen (University of Oslo, Norway) and cultured at 37 °C in 5 % CO_2_ in RPMI 1640 (Sigma-Aldrich, Rehovot, Israel) supplemented with 10 % Fetal Bovine Serum (FBS), 1 % MEM NEAA 100x (Gibco), 0.005 % 1 M I-thioglycerol, 0.03 % gentamycin 40 mg/ml (Sigma-Aldrich), and 2 mM L-glutamine (Biological Industries, Beit Haemek, Israel). To induce myeloma, 5000 logarithmic phase myeloma cells were injected into the tail vein of the mice at age 16–18 weeks (ELMM group). A group of age and weight-matched control mice were similarly inoculated with PBS vehicle [17,16]. At several time points over the next 220 days, BM and/or peripheral blood samples were collected from 4 to 6 mice in both the Control and ELMM groups and analyzed as described below. Samples were collected and coded blindly by qualified laboratory researchers.

### Determination of serum levels of paraprotein

Approximately every 70 days post-inoculation, tail vein blood was drawn, and the serum was frozen at −20 °C until used. Serum IgA levels were measured by ELISA. Briefly, 96 well Nunclon ELISA plates were coated with 5 µg/mL goat anti-mouse IgA (Southern Biotech) overnight at 4 °C. Wells were blocked with PBS/0.02 % sodium azide/1 % BSA, washed, and incubated for 1 h at 37 °C with serum samples or standard mouse IgA (ranging from 625 to 3.75 ng/ml) diluted in PBS/0.02 % sodium azide/0.1 % BSA/0.1 % Tween 20. Then, the plates were washed and incubated with goat anti-mouse HRP (Southern Biotech), incubated for 45 min at RT, and washed again. TMB substrate (Merck Millipore, Billerica, MA, USA) was added for 10 minutes, the reaction was terminated with H_2_SO_4_, and the absorbance was measured at 450 nm with a TECAN Infinite M200 ELISA reader.

### Behavioral tests (Hot plate test)

Previously, we demonstrated that limb functionality is a non-invasive surrogate of bone damage in myeloma-bearing mice [16]. Limb thermal hyperalgesia sensitivity was evaluated on day 190 and day 210 post-cell inoculation by gently placing the animal's hind legs on an aluminum plate maintained at 55 °C (Columbus Instruments, Columbus, OH). To prevent tissue damage, the latency of the paw reaction was measured with a cut-off of 40 seconds.

### Whole blood counts

Whole blood was drawn from the tail vein of myeloma-bearing and same-age controls every 30 days into EDTA-containing tubes and analyzed for hemoglobin, red blood cells, lymphocytes, granulocytes, monocytes and platelets were measured directly using an automatic veterinary hematology analyzer (Exigo H400 System®).

### Flow cytometry

Single-cell suspensions of BM mononuclear cells (BMMC) were prepared by flushing the femurs and tibias of mice with PBS. The eluant was treated with 1x RBC lysis buffer (eBioscience) for 3 minutes, centrifuged at 300 x g for 10 mins at 4 °C, and the cells resuspended in PBS to a concentration of 2 × 10^6^ cells/ml. Fc receptors were blocked by the addition of anti-CD16/CD32 antibodies (clone 93, eBioscience), and the cells were then stained for 30 min at RT in the dark with combinations of 19 fluorescently labeled anti-CD marker antibodies. These antibodies were divided into three panels designed to analyze different T cell subsets of T memory, T helper cells, NK, and DC cells (see Supplementary Tables S1, S2). Fluorescence was measured with a Cytoflex (eBiosciences) flow cytometer, and the data were analyzed with FlowJo software (TreeStar, Ashland, OR, USA). The gating strategy and compensation matrix for each panel included FMO controls. Gating schemes and antibodies are shown in Supplementary Tables S1-S2 and Figures S1-S3.

### In vitro T cell activation and cytotoxicity assays

BMMCs were collected as described. Target MOPC315.BM cells were treated for 24 hr with 5 µg/ml mitomycin C (Sigma-Aldrich) to arrest cell growth. Following washing, the BMMC and MOPC315.BM cells were added to culture dishes coated with 5 µg/mL anti-CD3 antibody and co-cultured for 4 days at a ratio of 20:1 (BMMC-to-target) in complete medium (see above) supplemented with soluble anti-CD28 (2 µg/mL) and recombinant mouse IL-2 (50 U/mL, Biolegend). CD8+ cells were purified from these co-cultures using CD8+ microbeads (Milteny) and tested for CD8 and CD69 positivity by flow cytometry. To test the cytotoxicity of these cells, 10^7^ MOPC315.BM cells were labeled with 1 μM CFSE (eBioscience) for 10 min at RT. The reaction was stopped by adding 4–5 volumes of cold complete media followed by 5-min incubation on ice. After washing, the labeled myeloma cells were resuspended in complete medium at 10^6^ cells/mL and dispensed into 96-well microtiter plates. The isolated CD8+ cells were then added to the labeled target cells at 20:1 effector-to-target ratio in a total volume of 250 µL complete medium, and the plates were incubated at 37  °C in 5 % CO_2_ for 4 hrs. The cells were then harvested and stained with anti-mouse interferon-gamma (IFN γ) labeled with PE, and anti-mouse CD44 PE/CY7 (BLG-103030) and LIVE/DEAD Fixable Red Dead stain 488 nm (Thermofisher Scientific). The degree of myeloma cell killing was calculated using the following formula:

%MOPC315.BMcellskilling=(%CFSE−labeledmyelomacellsalone−%CFSE−labeledmyelomacellsinthepresenceofeffectorcells)/%CFSE−labeledmyelomacellsalone.

Target cells incubated without effector cells were used as spontaneous cell death control. The data were acquired by a Cytoflex (Beckman Coulter) flow cytometer and analyzed using FlowJo 10.8.1 software.

### Statistics

Prior to comparing results between groups, the normal distribution of raw data for each group was validated using the Shapiro-Wilks test. Two-way ANOVA with the Bonferroni mixed comparison of Log2 transformed raw data was used to compare differences in between Control and ELMM groups, Fold Changes (FC) in individual ELMM mice/Mean Control at each time point, and Mean Fold Changes in ELMM mice over time. All statistical analyses were performed using GraphPad Prism 10.4.1 or 2 or Excel. Statistical significance between groups was determined at *p* < 0.05.

## Results

Following inoculation of mice with 5000 MOPC315.BM myeloma cells (ELMM mice), peripheral blood, and bone marrow samples were analyzed on days 80, 150, and 220.

### Changes in diagnostic parameters reflect the progression of myeloma disease

**Fig. 1:**
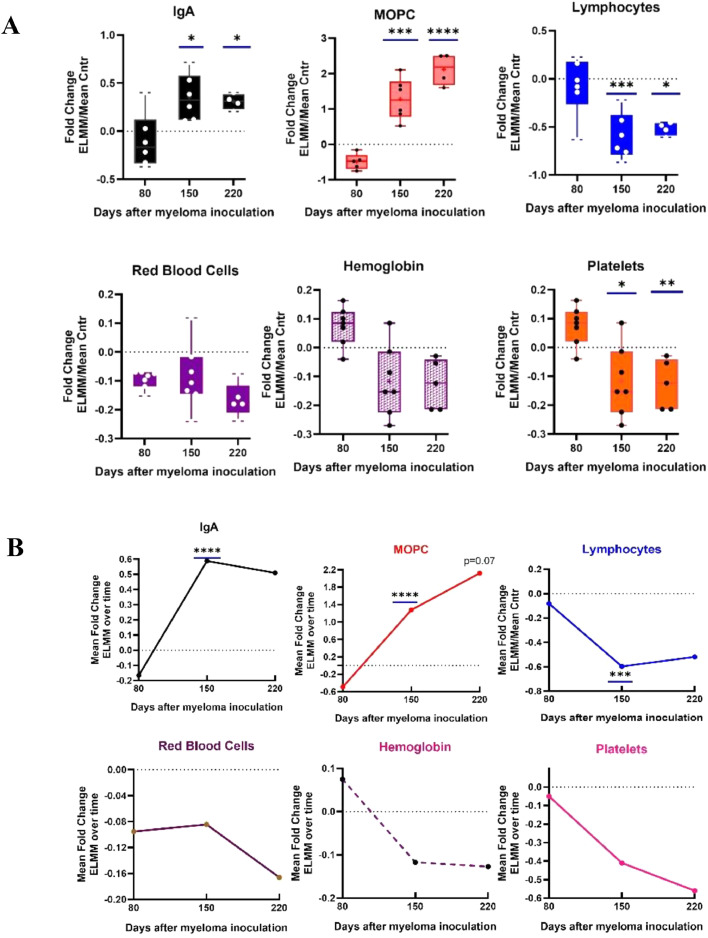
Time course measurements of hematological parameters depicting the development of myeloma in the ELMM mouse model. Balb/c mice were inoculated intravenously with 5000 monoclonal IgA paraprotein-producing MOPC315.BM cells (ELMM) or PBS vehicle (Control) and tail blood samples were collected at 3 time points up to 220 days after inoculation. Raw data were transformed into Log2. **A:** Each whisker box represents the Fold Charge between ELMM mice and the Mean of the Control group. **B**: Changes in normalized Fold Changes in each parameter over the course of myeloma development. Statistically significant differences (tested by 2-way ANOVA) are depicted by bars on top of the boxes *p ≤ 0.05; ** *p* ≤ 0.01; *** *p* ≤ 0.001; **** *p* ≤ 0.0001.

Direct comparisons of Log2 transformed raw data between ELMM and Control for several blood parameters are shown in Figure S4. To more accurately track changes in ELMM mice over time, Log 2 Fold Change)FC) between groups at each time point was analyzed for significance, as shown in Fig. 1A. As in human MM, disease development in myeloma-bearing mice was associated with significant elevation in serum paraprotein (MOPC315.BM cells secrete a monoclonal IgA antibody) from day 150 (day 150 *p* = 0.014, day 220 *p* = 0.05); significant proliferation of myeloma cells in the BM (day 150, *p* = 0.003; day 220, *p* < 0.0001); lymphopenia which was significant by day 150 (day 150 *p* = 0.009, day 220 *p* = 0.015); anemia characterized by decreases in red blood cells and hemoglobin by day 150, and a significant loss of platelets from day 150 (day 150 *p* = 0.011, day 220 *p* = 0.003). In contrast, the frequencies of granulocytes and monocytes were statistically similar in Control and ELMM mice throughout the experiment (data not shown). In addition, we previously reported that retarded limb mobility might serve as a non-invasive indicator of bone damage [16]. In the present study, the hot plate flight response was significantly slower in myeloma mice by Day 150 (*p* = 0.003) (data not shown).

Normalizing the Log 2 values for the ELMM mice against the mean Log 2 values for the Control group at each time point allowed tracking changes in each parameter in the ELMM group as disease developed. The results shown in Fig. 1B indicate that aside from Red Blood Cell counts, the systemic hematological consequences of early myeloma proliferation were apparent midway through disease progression, by Day 150.

### Changes in T cell subset profile in myeloma-bearing mice versus healthy controls

**Fig. 2:**
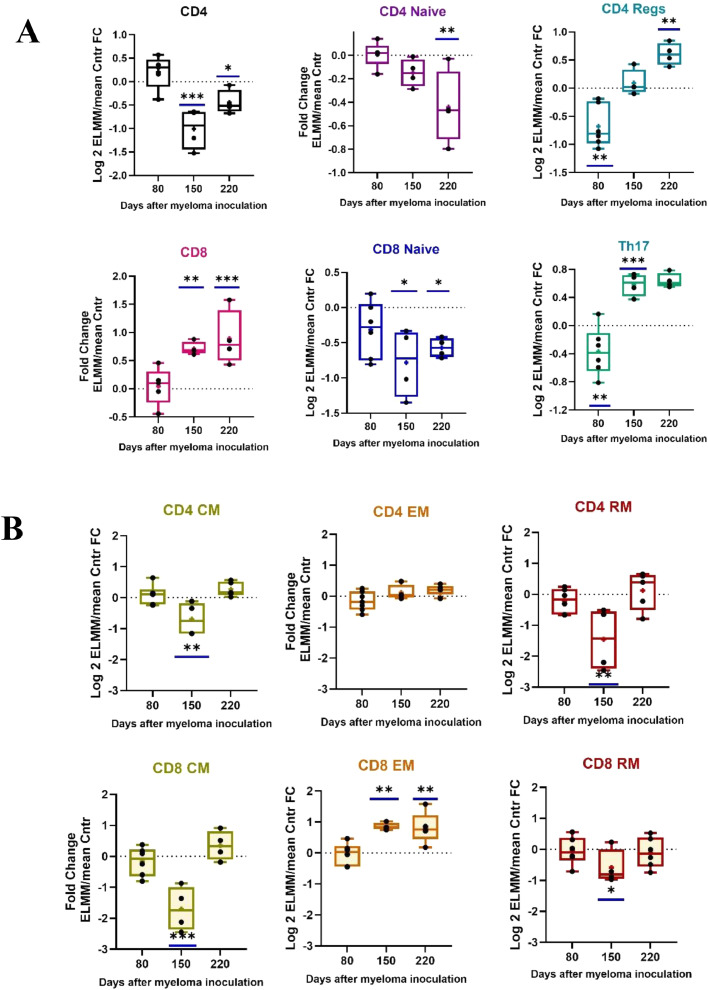
Tracking fold changes (FC) in T-cell sub-population frequencies in E-LMM over Control mice during the development of myeloma. Bone Marrow-derived T-cells subsets were stained with fluorochrome-labeled antibodies and analyzed by flow cytometry. The data were processed with FlowJo software according to the gating strategy depicted in Figure S1-S3. % frequencies were transformed into Log2, and the FC differences were tested for statistical significance (tested by 2-way ANOVA). FC values for each group are presented as whisker boxplots. **A**) Total CD4+, and CD8+ cells in the CD3+ fraction; Naïve cells in the CD4+ or CD8+ fraction; T-regulatory and Th17+ cells in the CD4+ fraction. **B)** Central Memory, Effector Memory and Resident Memory cells in the CD4+ and CD8+ fractions. Statistically significant changes in ELMM levels are depicted by bars on top of(ELMM are higher) or below (ELMM levels are lower) of the boxes *p ≤ 0.05; ** *p* ≤ 0.01; *** *p* ≤ 0.001; **** *p* ≤ 0.0001.

The % frequency of CD4+, CD4+ Naïve, CD4+ Reg, Th17, CD8+ and CD8+ Naïve T cell subsets in Control and ELMM groups at each time point is shown in Figures S5. These frequencies were Log2 transformed, and the Fold Changes (FC) between the ELMM mice and the mean of the Control group at each time point was plotted as shown in Fig. 2A (bars with asterisks depict the degree of significant difference). The FC in frequency of total BM-derived CD4+ were normal at day 80 following inoculation but dropped significantly by day 150 (*p* = 0.003) and remained below normal at day 220 (*p* = 0.04). The opposite was true for total CD8+ cells whose levels progressively elevated above normal at day 150 (*p* = 0.004) and day 220 (*p* = 0.008). The presence of CD4+ and CD8+ Naïve T-cells (TN) decreased significantly as the disease progressed (day 220: CD4+, *p* = 0.0015, CD8+ *p* = 0.015). Interestingly, the level of both CD4+ Tregs and Th17+ cells were lower than controls in the early stage (Tregs *p* = 0.001, Th17+ *p* = 0.023) but became very abundant as the disease progressed (Tregs *p* = 0.006, Th17+ *p* = 0.0011).

Dissection of T memory cells showed that the BM frequencies of some subsets =*n* ELMM mice were disrupted by day 150 (Fig. 2B). By this time both CD4+ and CD8+ T-central memory (TCM) cells had significantly decreased below normal levels (CD4+TCM *p* = 0.003; CD8+ TCM *p* < 0.001), but by day 220, both had returned to those of the controls. A similar pattern was seen for both CD4+ and CD8+ T resident memory (TRM) FCs. Regarding T- effector memory (TEM) cells, CD4+ levels remained similar to controls throughout the observation period, while CD8+ FCs has risen significantly by day 150 (*p* = 0.003) and remained so at day 220.

### Relative changes in T cell profiles in myeloma-bearing mice during disease progression

**Fig. 3:**
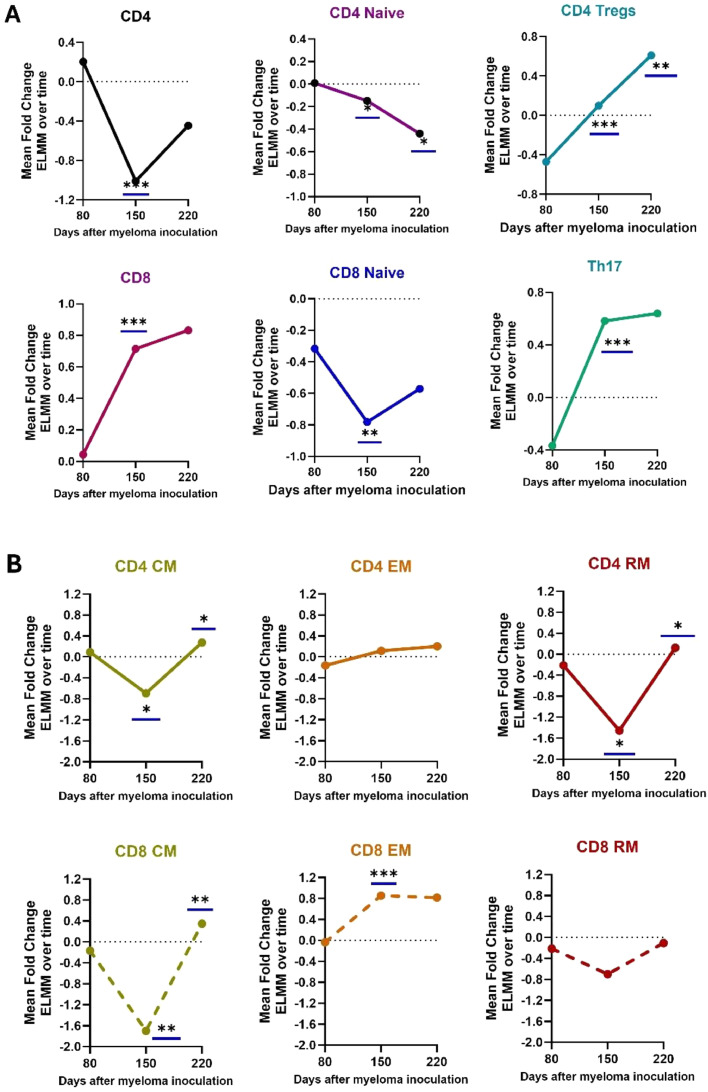
Tracking relative fold changes (FC) in T-cell sub-populations in E-LMM mice during development of myeloma. Bone marrow-derived T-cell subsets were stained with fluorochromelabeled antibodies and analyzed by flow cytometry. The data were processed with FlowJo software according to the gating strategy depicted in Figure S1-S3. The normalized Log2FCs for E-LMM mice at each time point were used to compare changes in FCs as the disease developed. CM, Central Memory, EM, Effector Memory and RM, Resident Memory cells. Significant differences in FCs between time points, (identified by one-way student t-test with equal or unequal variance as determined by the F-test) are shown. *p ≤ 0.05; ** *p* ≤ 0.01; *** *p* ≤ 0.001; **** *p* ≤ 0.0001. See text for actual P values. **(C)** Relative changes in % between TN, TCM, TEM, and TRM cells from days 80-220.

We then asked whether the model detects changes in the relative frequency of each T cell subset as myeloma develops. To answer this question, the normalized Log2 ELMM Fold Changes were compared at each time point. The results shown in Fig. 3A demonstrate progressive but variable alterations in immune profiles as the disease progresses. Whereas the presence of Total CD4+ cells in ELMM mice fell dramatically by day 150 (*p* < 0.001), and remained low at day 220, the Total CD8+ population increased significantly at each time point (day 80-150, *p* = 0.004;80-220, *p* = 0.004). Parallel to the expansion in CD8+ cells, the levels of both Tregs and Th17+ cells (which were significantly lower than controls in early-stage disease) also increased significantly as the disease progressed (Tregs: day 80-220, *p* = 0.002, day 150-220, *p* = 0.008: Th17+ day 80-150, *p* = 0.0002).

Regarding relative fold changes in Naïve and memory T cell subsets in the ELMM mice (Fig. 3B), the normalized FCs of both CD4+ and CD8+ TN cells decreased by day 150 (CD4+, *p* = 0.04; CD8+, *p* = 0.015). CD4+ TN declined further in the later stage of disease (day 220, *p* = 0.03) while CD8+ cells improved but were still below normal levels. Interestingly, CD4+ and CD8+ TCM and TRM cell levels dropped, usually significantly, in mid-stage (day 150) but returned to control levels by day 220. In contrast, CD4+ TEM cells remained similar to controls through the observation period, while their CD8+ counterparts rose significantly in mid-stage of the disease (day 150, *p* = 0.0005) and remained high.

Overall, the profile of relative changes in T-cell subsets from day 80 to day 220 suggests that phenotypically, the BM in later-stage MM disease is characterized by the expansion of CD8+ T cells, mainly of the TEM subset, but also increased CD4+ Tregs and Th17+ cells.

### CD8+ T cells are functionally exhausted early in disease progression

**Fig. 4:**
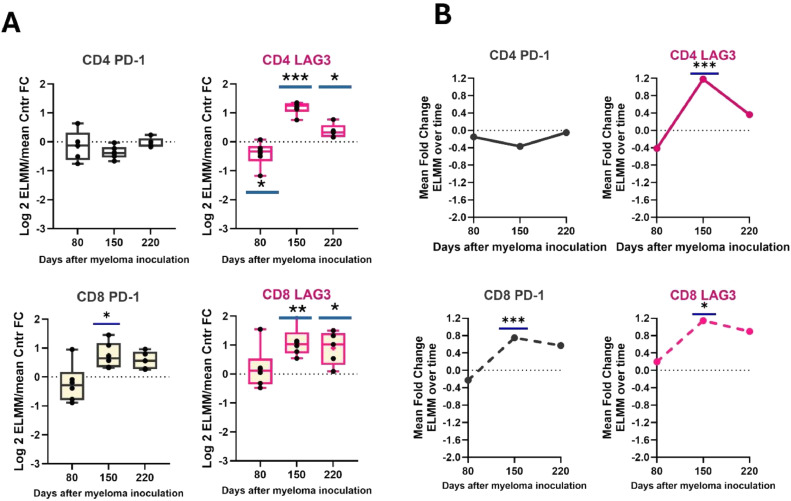
Changes in frequency of exhausted bone marrow T cell subsets in myeloma-bearing mice (E-LMM) as they develop disease. **A**) Immunophenotyping of CD4+ and CD8+ *T* cells demonstrates a significant rise in LAG3+ *T* cells and in CD8+ PD-1+ cells from ELMM over the Control group mice, **B)** as well as a significant rise in these cells in the ELMM group over time. Significant differences in ELMM/Control fold changes between time points. *p < 0.05; ** *p* < 0.01; *** *p* < 0.001.

Given the apparent dichotomy between the increased frequencies in total CD8+ cells, TEM cells and Tregs as the disease develops, we next asked whether the CD8+ cells were functional. Indications that this was not the case were that these cells showed clear signs of exhaustion, as indicated by the significantly enhanced expression of LAG3 on both CD4+ and CD8+ cells over controls on days 150 and 220 (CD4+ *p* = 0.0001 and *p* = 0.04; CD8+ *p* = 0.006 and *p* = 0.03, respectively) (Fig. 4A). Interestingly, CD4+LAG3 levels were lower than those of controls at day 80 (*p* = 0.04). Although CD4+ PD-1 levels were similar to controls, PD-1 expression on CD8+ cells were significantly elevated at day 150 (*p* = 0.02) and remained above controls at day 220. Similar changes were seen when the normalized fold change of ELMM mice between time points was compared (Fig. 4B).

**Fig. 5:**
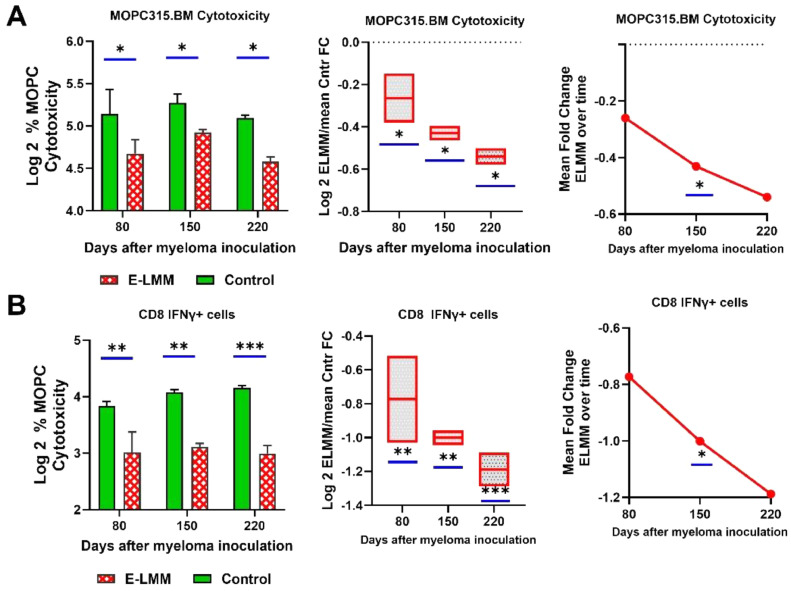
**C**ytotoxic capacity of bone marrow-derived CD8+ *T* cells from ELMM and control groups. **A**) Bone-marrow-derived CD8+ *T* cells were cultured with CSFE-labeled MOPC315.BM myeloma cells. The decrease in the CSFE-positive cell fraction as measured by flow cytometry was used to define the % Myeloma cell killing. **B)** The level of IFNγ+ within the CD8+ *T* cells were also measured over time. The results show a significant decrease in CD8+ myeloma cytotoxicity as disease develops. Statistical significances were calculated by using 2-way ANOVA (left and middle panels) and the onetailed t-test with Welch’s correction. *p < 0.05, **p < 0.01, ***P < 0.0001.

To directly assess the anti-myeloma functionality of CD8+ T-cells, we tested the ability of BM-derived CD8+ T cells from healthy or myeloma-bearing mice to kill MOPC315.BM cells *in vitro* and to secrete IFNγ. Fig. 5A demonstrates that the cytotoxic capacity of CD8+ T-cells from myeloma-bearing mice was already significantly decreased by day 80 (*p* = 0.018) and remained so throughout the experiment (day 150, *p* = 0.05; day 220, *p* = 0.012). The normalized Fold changes in ELMM mice also significantly decreased over time. The decreased functional cytotoxicity was characterized by a significant drop in the appearance of IFNγ producing CD8+ T cells from ELMM mice at each time point (Fig. 5B).

### Frequencies of BM-derived dendritic cells and natural killer cells increase during the development of MM

**Fig. 6:**
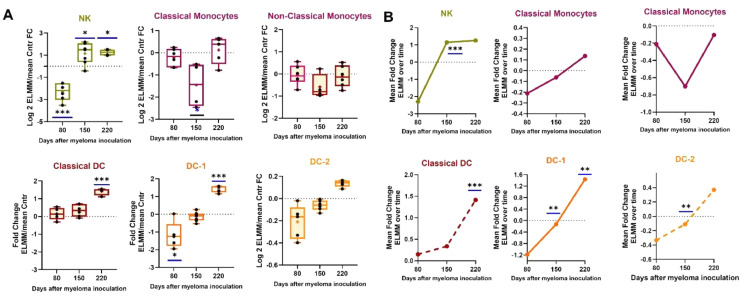
Changes in frequency of bone marrow-derived Natural Killer (NK), Classical Dendritic cells (Classical DC), DC subsets, DC-1 and DC-2, Classical and non-Classical Monocytes in myelomabearing mice (ELMM) as they develop disease. The frequencies for each ELMM mouse at each time were normalized by calculating the Log2 fold change (Log2FC) of each data point over the mean frequency of the Control group for that time point. A) Mean Log2FC in ELMM over Controls on days 80, 150, and 220 post-myeloma inoculation. B) Changes in mean ELMM frequencies over time of the experiment. *p < 0.05; ** *p* < 0.01; *** *p* < 0.001.

The involvement of NK and DC cells during the disease's development was also tracked compared to healthy controls (Fig. 6). At the early time point, NK cells were significantly lower than in controls (*p* < 0.0001), but then the levels recovered to above normal (day 150, *p* = 0.012). For dendritic cells, frequencies are elevated as the disease develops, significantly so in the case of classical and DC-2 cells. There were almost no significant alterations in monocyte levels, except for a reduction in Classical monocytes at the middle time point (*p* = 0.5).

## Discussion

The natural history and underlying mechanisms that govern the breakdown in immunosurveillance leading to the development of active MM remain largely unresolved [18,19]. Acquiring the information to solve these issues from longitudinal patient studies has yet to be achieved, mainly due to the logistic limitations of following the same patients over several decades as they progress from pre-malignant to clinically active MM. Therefore, to promote further studies on the pathogenesis and therapy of MM, we aimed to recapitulate the natural history of the human disease in a murine model by inoculating a small number of MOPC315.BM myeloma cells were placed into immunocompetent Balb/c mice, and their BM cellular immune response and blood parameters were monitored for 220 days post-inoculation. This protocol was defined based on calibration experiments reported elsewhere [16]. Other murine models of myeloma have been developed in immunocompetent mice (reviewed in [20], however, to our knowledge, this is the first report of a long-term model in non-genetically manipulated, immunocompetent mice that parallels human MM and provides a tool for in-depth study of immune functional alterations from very early to advanced disease.

Several points emerge from our study. First, the ELMM model reflects the biochemical and hematological parameters seen in MM patients at diagnosis, such as paraproteinemia, lymphopenia, anemia, thrombocytopenia (Fig. 1), and bone lesions.

Second, the model can help resolve questions about the immunology of MM, which have direct relevance to the development of more effective therapies. An example of this is the still unsettled role of CD4+ Tregs in early-stage, treatment-naive MM, with different studies reporting similar [21,22] increased [23,12], or decreased [24,25] frequencies when compared to healthy controls. These discrepancies may be due to methodological or population differences, but they emphasize the difficulty in comparing results between clinical studies on different groups of patients. Our model clearly shows that when compared directly to healthy animals or when normalized and tracked for changes over time, the levels of CD4+ T regs in ELMM mice appear to be biphasic (Figs. 2A and 3A), decreasing in the early stages of the disease and then rising dramatically as the disease develops. Moreover, this biphasic pattern was also seen for both Th17+ cells and total CD8+ cells, paralleling myeloma cell proliferation (Fig. 2A). In contrast, total CD4+ levels decreased over time. These results may seem contradictory initially, as instinctively, Tregs are recognized as immunosuppressive of CD8+ T cell anti-tumor immunity, and CD4+ Th17+ T-cells are pro-inflammatory [26]. However, this is exactly the picture that has emerged from bioinformatic and flow cytometric studies on patients with MGUS, SMM, and newly diagnosed MM [27,28]. Indeed, our model describes the changing microenvironment of the BM as the disease progresses, as noted by others [27]. Based on these data, we suggest that the initial low levels of Tregs and Th17+ cells may offer a window of opportunity for a CD4+ T-cell-assisted CD8+ T cell cytotoxic immune attack [29,30]. This initial immune response occurs very early when serological and biochemical parameters associated with MM appear normal (Fig. 1). Although the initial interactions between the myeloma clone and stromal and immune elements in the BM remain to be elucidated [27], failure of this initial response would afford the continued interplay between the myeloma niche and surrounding BM components, either by direct contact or through the skewed effects of their secreted growth factors and cytokines, eventually leading to immune suppression and promotion of myeloma cell proliferation [31]. Support for this scenario comes from direct studies showing a connection between increased levels of Tregs and Th17+ cells, myeloma cell proliferation, and accelerated bone destruction [32,33].

The data on T memory cells also fits the above proposition. The success of anti-tumor T cell-mediated immune responses is in large part dependent on the pathways taken by TN cells as they differentiate into effector or regulatory T cells following antigenic activation [34]. The differentiation pathway also influences the dynamic relationship between Tregs and effector T-cell function. Unlike the transitory stimulation seen during an infection, the continual T cell activation by tumor antigens makes it difficult to separate and code the relative contributions of various T cell subsets to cancer immunity [35]. Indeed, while several studies demonstrate that cytotoxic CD8+ effector T cells are essential for the destruction of tumor cells [36,37], the influence of the major subsets of T memory cells, TCM, TEM, and TRM on this response is still an emerging field of study [37,34,38] . This is especially so in MM, where information is lacking on their respective roles in anti-myeloma immunity as the disease progresses.

The characteristics of these cells have recently been reviewed [39,36]. Classically, TCM cells primarily reside in lymph nodes, but can circulate to other tissues. Antigenic stimulation leads to their high proliferation and longer survival but relatively low immediate effector function, suggesting they may be more critical for maintaining a lower-level anti-tumor response. TRMs play a role in immunosurveillance and act as initial responders to cellular antigens. This classical pattern of T memory cell behavior becomes disturbed as MM progresses. Notably, fold changes in both TCMs and TRMs in ELMM mice almost mirror those for Tregs (Figs. 2B and 3B), dropping significantly at the intermediate stage of the disease, further highlighting the immunosuppression that takes hold during this time. The third classical subset of T memory cells, TEMs, are circulatory and, upon infiltration into tumor tissue, can have contrary effects on tumors. They can mediate tumor progression by skewing the tumor-immune equilibrium through cytokine secretion, but alternatively, the CD8+ fraction of TEMs possesses lytic capacity [40]. Our results also point to the complex nature of these cells. While Figs. 2B and 3B show that the CD4+TEM subset was essentially unaffected by the disease, CD8+ TEM cells rose significantly as the disease developed. This increase is in concert with the overall rise in CD8+ cells and may suggest the mounting of an aggressive, lytic anti-myeloma response. However, despite the increased presence of CD8+ *T* cells and the TEM subset, the immunosuppressive conditions in the BM lead to phenotypic signs of T cell exhaustion and cytotoxic dysfunction (Fig. 4, Fig. 5). These results parallel findings in patients with MM [41,14].

The involvement of innate immune cells such as NK and dendritic cells and their interaction with adaptive immune cells is complex, as these interactions result in the release of various cytokines whose interplay can lead to tumor deletion or progression [42,43]. Several studies have highlighted the intricate and complex crosstalk between NK and T-cells in the tumor microenvironment. The elevation over time in NK levels may be understood as an attempt by the innate immune system to compensate for the T-cell exhaustion and loss of CD8+ T-cell mediated tumor cytotoxicity as the disease progresses [43]. This explanation is also in line with the observed increase in DCs over time, as there exists bi-directional crosstalk between NK and DCs during immune responses [44]. However, NK cells can also contribute to immunosuppression by coordinating with Tregs [45], and Type-1 classical DCs have been shown to support myeloma progression [46]. Further studies are required to clarify these issues.

One limitation of our study may be the use of mature MOPC315.BM myeloma cells for which the mutational landscape has not yet been defined. However, support for their use comes from recent studies using both whole-genome as well as single-cell RNA sequencing (sc-RNA-seq), which indicate that the cytogenetic, mutational, and rearrangement profiles of myeloma cells in patients with SMM are very similar to those described in mature MM [47,48]. Furthermore, a very recent paper in this journal by the De Veirman group [49], describes a comprehensive sc-RNA-seq and flow cytometry study of the 5T33MM mouse myeloma model using a more aggressive, short-term protocol of just 20 days, akin to our previously reported short-term MOPC315.BM MM model reported earlier [17]. The de Veirman study also reported progressive immunosuppression and elevation in TEM cells that were exhausted and dysfunctional in their MM-lytic capacity. Importantly, these alterations in immune cellular immune BM occupancy and function were also reported in a recent scRNA-seq analysis of the BM from patients with MGUS, SMM, or newly diagnosed MM [50], suggesting that our model reflects the immunological, behavioral, and hematological characteristics of human MM disease progression.

As questions remain about the initial cellular interactions that initiate immunosuppression and MM disease progression, our model is useful for investigating these queries. The data demonstrates a crucial early challenge to immunosurveillance by the presence of myeloma cells which is not reflected in current diagnostic tests for MM, highlighting the need to identify new biomarkers of this immune response failure. Such biomarkers may help to stratify individuals eligible for therapeutic intervention at the precursor stages of disease with either T-cell redirecting strategies such as CAR-T cells (reviewed in [5]) or with strategies to revitalize exhausted CD8+ T cells by manipulating the microbiome or directly using gut microbial products such as short chain fatty acids.

## Consent for publication

All authors have given consent for publication.

## Funding

SJ, VS and AL were supported by graduate fellowships from Ariel University. This research was funded by the Ariel University Research Authority.

## CRediT authorship contribution statement

**Suchita Suryakant Jadhav:** Investigation, Writing – original draft, Conceptualization, Formal analysis, Methodology, Data curation. **Vipin Sharma:** Investigation, Formal analysis, Methodology. **Aharon Lion:** Formal analysis, Methodology, Investigation. **Lasser-Katz Efrat:** Methodology. **Iftach Shaked:** Formal analysis, Writing – original draft, Conceptualization, Resources. **Galia Luboshits:** Methodology, Writing – original draft, Resources, Conceptualization. **Michael A. Firer:** Formal analysis, Conceptualization, Funding acquisition, Writing – original draft, Resources, Writing – review & editing.

## Declaration of competing interest

The authors declare that they have no known competing financial interests or personal relationships that could have appeared to influence the work reported in this paper.

## Appendix Supplementary materials

Image, application 1
