# Supplementary material for: Biphasic behavior of T cell subsets reflects failure of early anti-myeloma response and leads to progressive T cell dysfunction
Source: Neoplasia. 2025 Jul 11;67:101208. doi: 10.1016/j.neo.2025.101208 (PMC12276441; doi:10.1016/j.neo.2025.101208)
Supplement: Supplementary file 1 [file mmc1.docx]

**Supplementary information**

## Table S1. Panels Antibodies conjugated fluorophores, clones, and vendor information

| Antibody | Fluorophore | Clone | Vendor |
| --- | --- | --- | --- |
| CD3 | APC/CY7 | 145-2011 | Biolegend |
| CD4 | BV605 | RM4-5 | Biolegend |
| CD8 | BV785 | 53-6.7 | Biolegend |
| CD25 | BV510 | PC61 | Biolegend |
| CD127 | Percp/PC5.5 | SB/199 | Biolegend |
| CD44 | PECY7 | IM7 | Biolegend |
| CD62L | AF488 | MEL-14 | Biolegend |
| CD69 | APC | HI.2F3 | Biolegend |
| CD103 | PE | 2E7 | Biolegend |
| PD1/CD279 | PE | 29F-1A12 | Biolegend |
| LAG3/CD223 | Percp/PC5.5 | C9B7W | Biolegend |
| CD183 | AF488 | CXCR3-173 | Biolegend |
| CD196 | PE CY7 | 29-1L17 | Biolegend |
| B220 | PE CY7 | RA3-6B2 | Biolegend |
| CD138 | APC | REA104 | Milteny Biotech |
| CD11C | FITC | N418 | Biolegend |
| CD49B | AF700 | DX5 | Invitrogen |
| CD355 | BV510 | 29A1.4 | Biolegend |
| Ly6C | PERCY 5.5 | HK1.4 | Biolegend |
| CD3 | Unconjugated | 17A2 | Biolegend |
| CD28 | Unconjugated | 37.51 | Biolegend |

Table S 2. Gating strategy to immunophenotyping

|  | Phenotype | Marker expression |
| --- | --- | --- |
| Panel 1 | CD4+ T naïve | CD3+/CD4+/CD44-CD62L+ |
|  | CD4+ TCM | CD3+/CD4+/CD44+CD62L+ |
|  | CD4+ TEM | CD3+/CD4+/CD44+CD62L- |
|  | CD4+ TRM | CD3+/CD4+/CD44+CD69**^+/-^** CD103+ |
|  | CD4+ Treg | CD3+/CD4+/CD25+CD127- |
|  | CD8+ T naïve | CD3+/CD8+/CD44-CD62L+ |
|  | CD8+ TCM | CD3+/CD8+/CD44+CD62L+ |
|  | CD8+ TEM | CD3+/CD8+/CD44+CD62L- |
|  | CD8+ TRM | CD3+/CD8+/CD44+CD69**^+/-^** CD103+ |
|  |  |  |
| Panel 2 | TH17 | CD3+/CD4+/CXCR3+ CCR6+ |
|  | CD4+T exhausted | CD3+/CD4+/PD1^+/-^ LAG3^+/-^ |
|  | CD8+T exhausted | CD3+/CD8+/PD1^+/-^ LAG3^+/-^ |
|  |  |  |
| Panel 3 | Total Conventional DC | CD3-/B220-/CD11C+ |
|  | CDC 1 | CD3-/B220-/CD11C+ CD8+ |
|  | CDC 2 | CD3-/B220-/CD11C+ CD8- |
|  | Plasmacytoid DC | CD3-/B220+/CD11C+ LY6C+ |
|  | NK | CD3-/B220-/CD49b+ CD355+ |
|  | MM | CD3-/B220-CD138+ |

## Figure S 1: Panel 1: Gating strategy to analysed different T cells subs


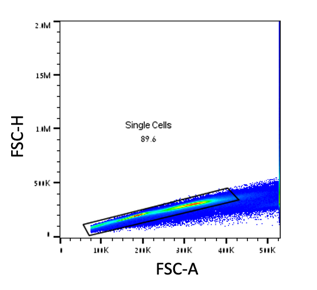

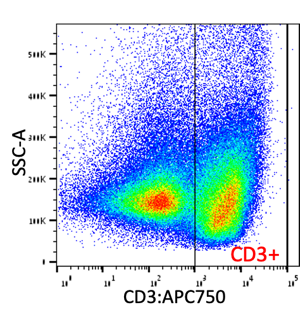

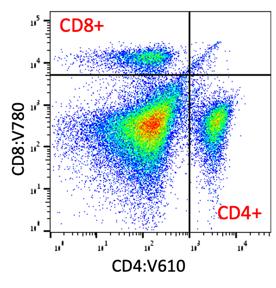

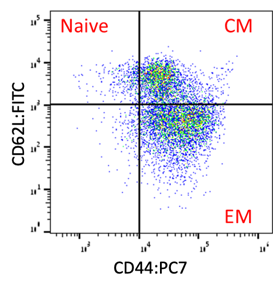

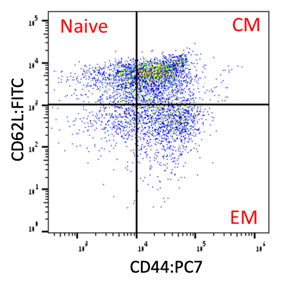

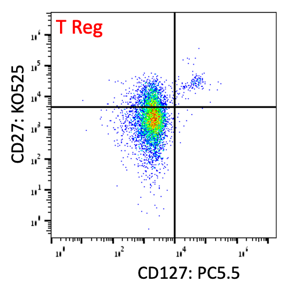

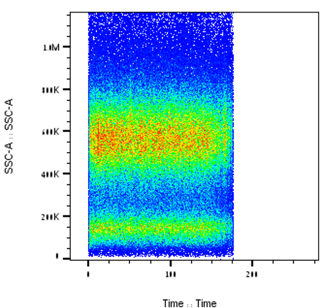

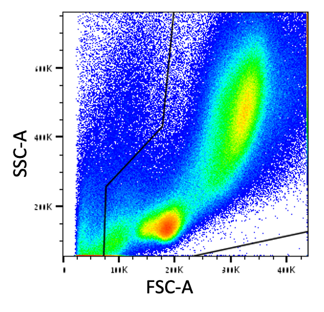

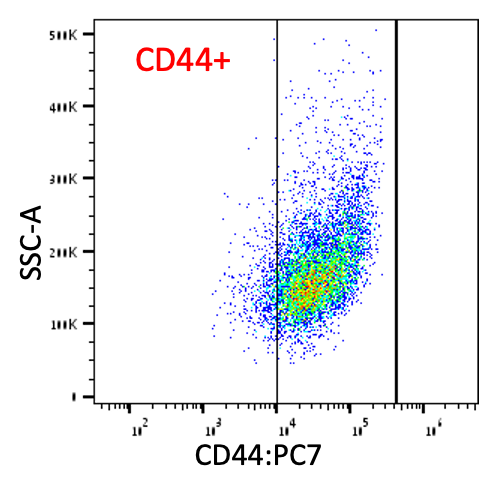

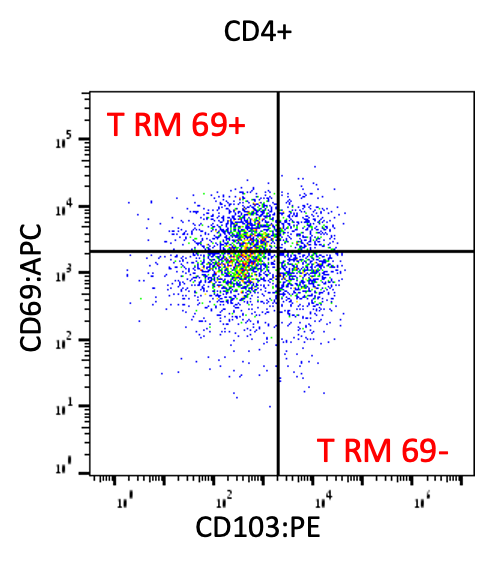

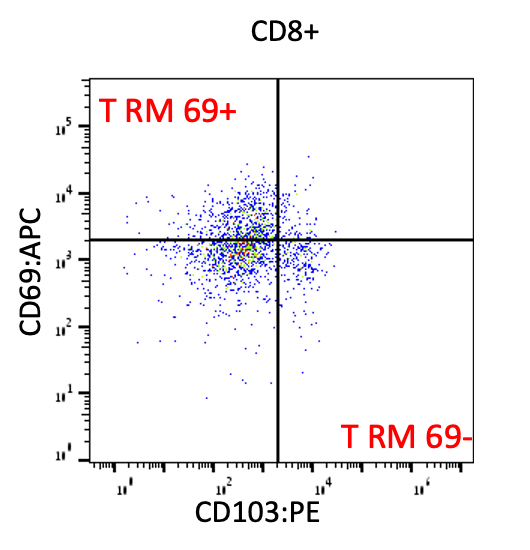


## Figure S 2: Panel 2: Gating strategy to analysed different T helper and exhausted cells


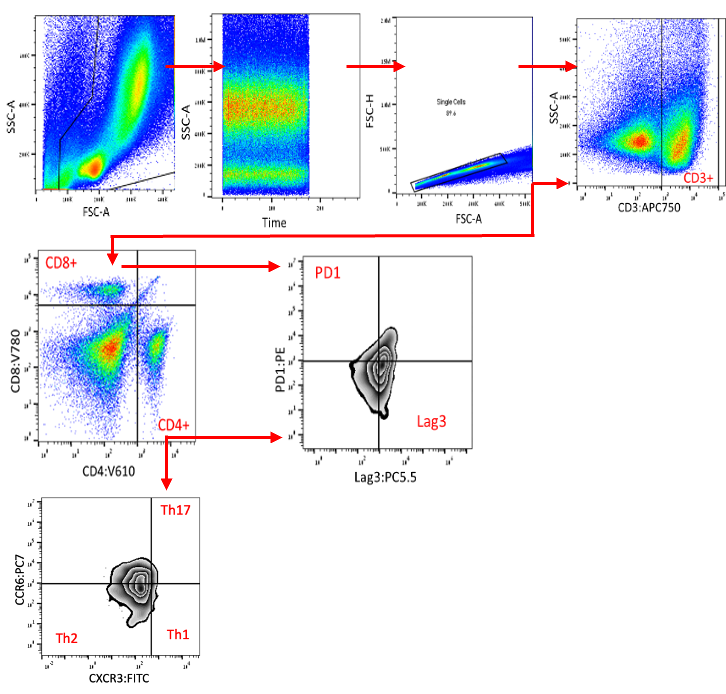


## Figure S 3: Panel 3: Gating strategy to analysed different subsets of DC and NK cells


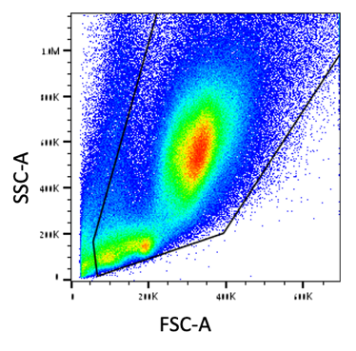

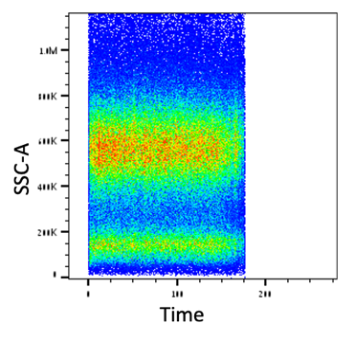

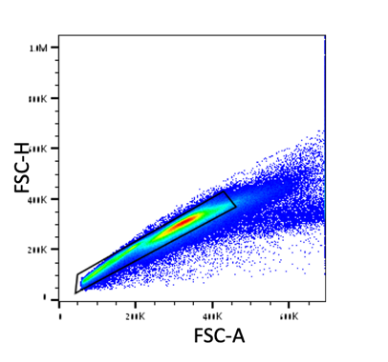

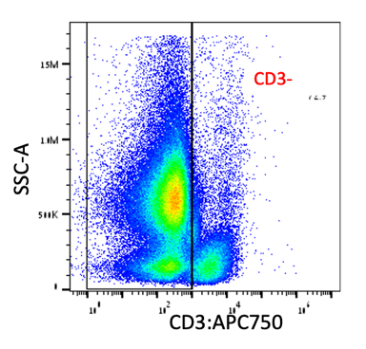

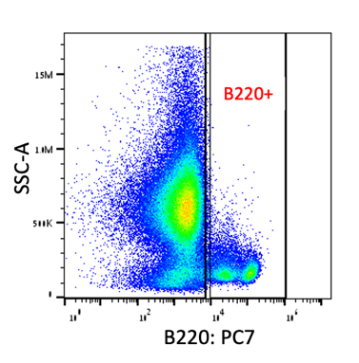

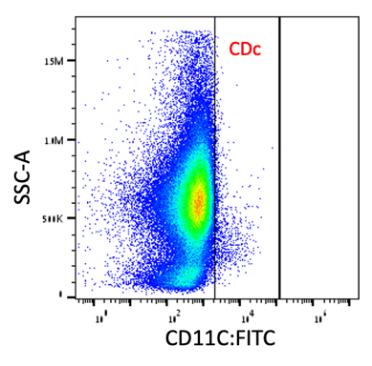

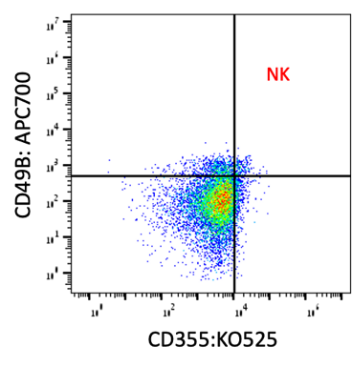


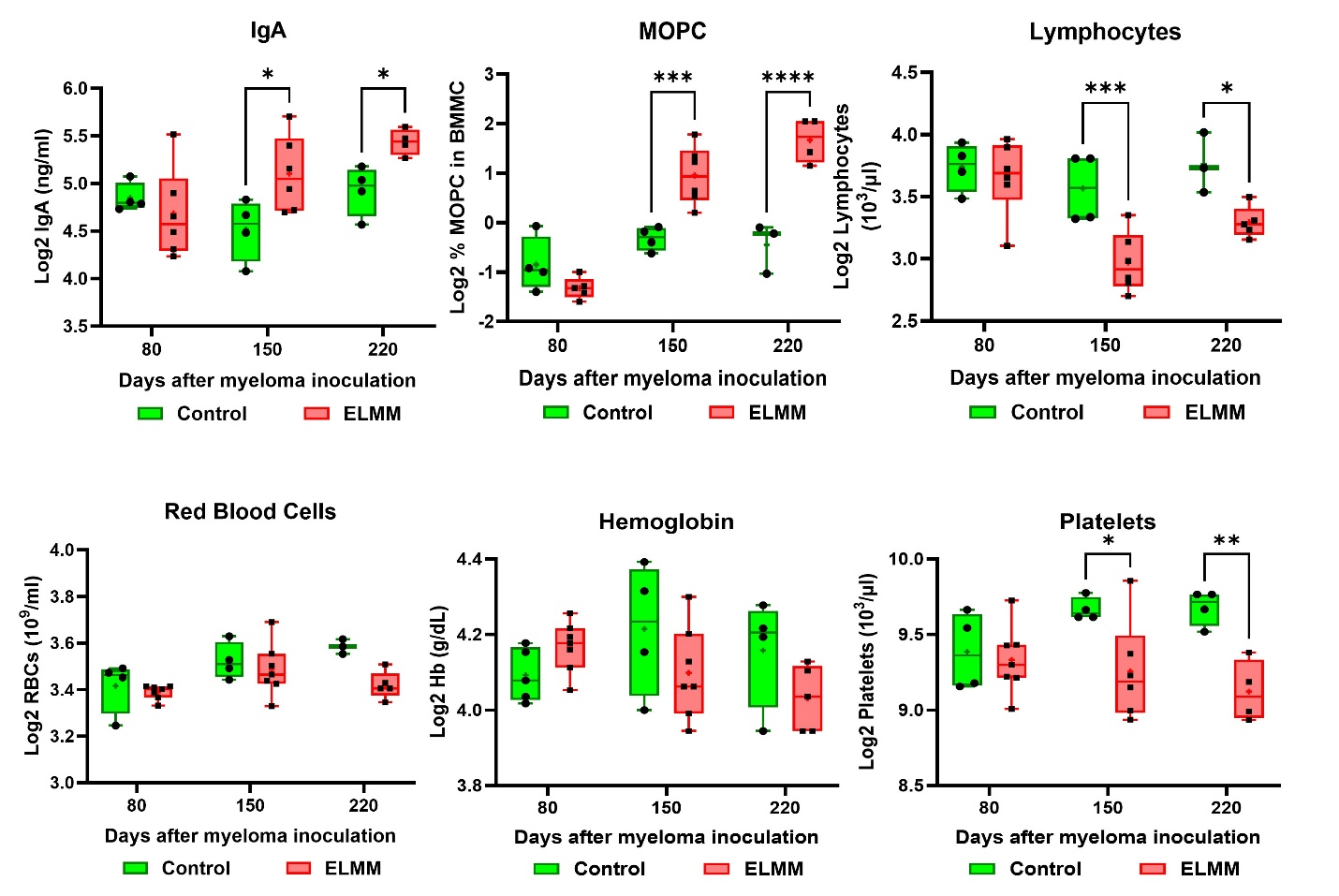


**Figure S4**: Serological parameters measured in Control and myeloma-bearing mice during development of myeloma in the ELMM model. ELISA measured Paraprotein (IgA) serum levels, while hemoglobin, red blood cells, lymphocytes, and platelets were measured directly using an automatic veterinary hematology analyzer (Exigo H400 System®). Values measured in the Control and ELMM groups at the three time points indicated following myeloma inoculation were Log 2 transformed and compared using 2-way ANOVA. Significant differences are indicated: *, p ≤ 0.05; **, p ≤ 0.01: *** p ≤ 0.001; ****, p ≤ 0.0001.


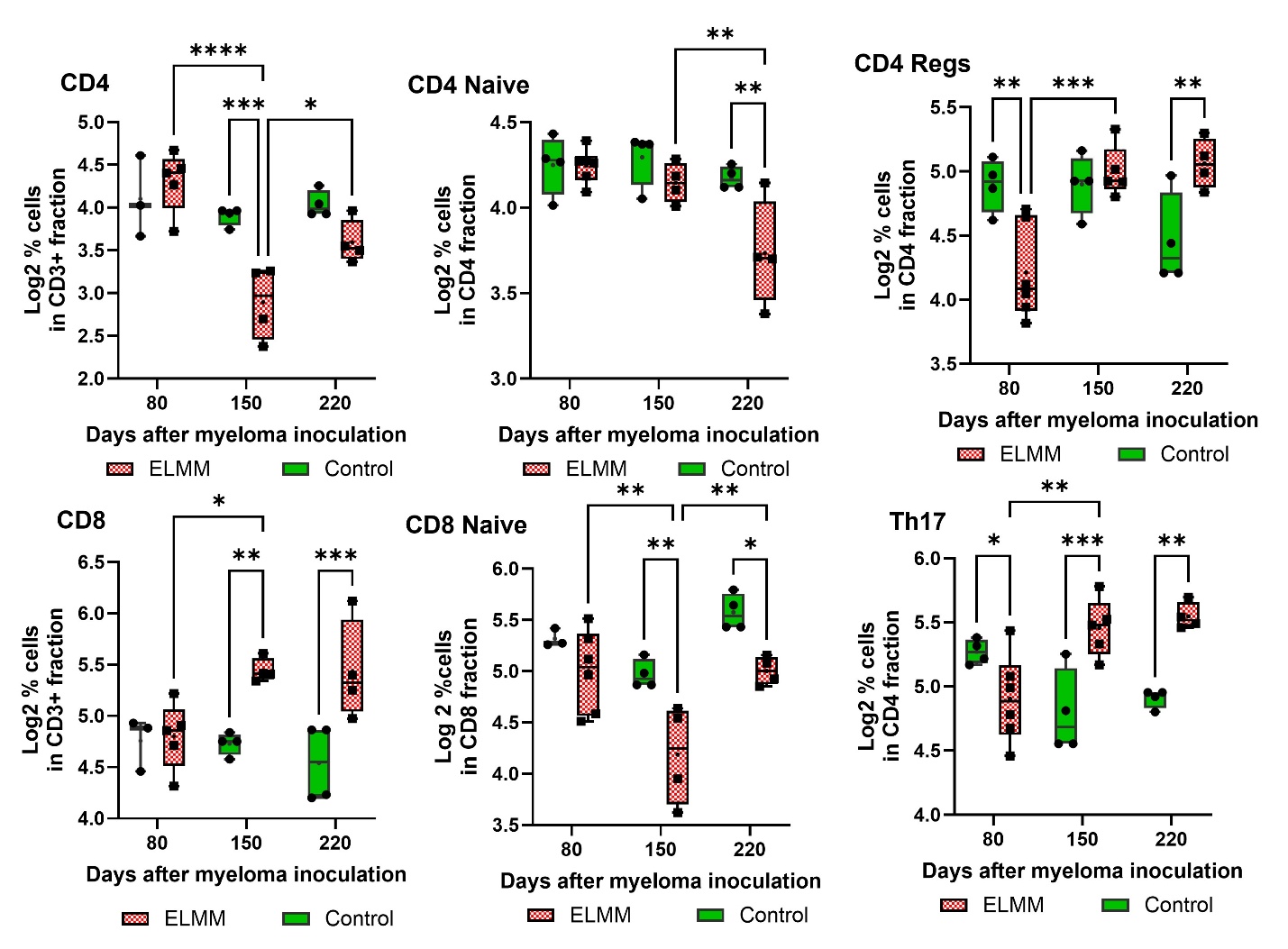


**Figure S5:** Tracking changes in T-cell sub-population frequencies in ELMM and Control mice during development of myeloma. Bone Marrow-derived T-cells were stained with fluorochrome-labeled antibodies and analyzed by flow cytometry. The data were processed with FlowJo software and are presented as whisker boxplots. **A**) Total CD4+, and CD8+ cells in the CD3+ fraction; Naïve cells in the CD4+ or CD8+ fractions; T-regulatory and Th17+ cells in the CD4+ fraction. **B)** Central Memory, Effector Memory and Resident Memory cells in the CD4+ and CD8+ fractions. Two-way ANOVA with the Bonferroni mixed comparison test was used to identify statistical differences in frequencies between Control) and E-LMM groups, *p < 0.05; ** p < 0.01; *** p < 0.001.
